# Metformin Attenuates Silica-Induced Pulmonary Fibrosis by Activating Autophagy *via* the AMPK-mTOR Signaling Pathway

**DOI:** 10.3389/fphar.2021.719589

**Published:** 2021-08-09

**Authors:** Shu-xian Li, Chao Li, Xin-ru Pang, Juan Zhang, Gong-chang Yu, Abrey J. Yeo, Martin F. Lavin, Hua Shao, Qiang Jia, Cheng Peng

**Affiliations:** ^1^ Shandong Academy of Occupational Health and Occupational Medicine, Shandong First Medical University and Shandong Academy of Medical Sciences, Jinan, China; ^2^ Neck-Shoulder and Lumbocrural Pain Hospital of Shandong First Medical University, Shandong First Medical University and Shandong Academy of Medical Sciences, Jinan, China; ^3^ University of Queensland Centre for Clinical Research (UQCCR), Brisbane, QLD, Australia; ^4^ Queensland Alliance for Environmental Health Sciences (QAEHS), The University of Queensland, Brisbane, QLD, Australia

**Keywords:** silica, metformin, pulmonary fibrosis, AMPK-mTOR, autophagy

## Abstract

Long-term exposure to crystalline silica particles leads to silicosis characterized by persistent inflammation and progressive fibrosis in the lung. So far, there is no specific treatment to cure the disease other than supportive care. In this study, we examined the effects of metformin, a prescribed drug for type || diabetes on silicosis and explored the possible mechanisms in an established rat silicosis model *in vivo*, and an *in vitro* co-cultured model containing human macrophages cells (THP-1) and human bronchial epithelial cells (HBEC). Our results showed that metformin significantly alleviated the inflammation and fibrosis of lung tissues of rats exposed to silica particles. Metformin significantly reduced silica particle-induced inflammatory cytokines including transforming growth factor-β1 (TGF-β1), tumor necrosis factor-α (TNF-α) and interleukin-1β (IL-1β) in rat lung tissue and HBEC culture supernatant. The protein levels of Vimentin and α-smooth muscle actin (α-SMA) were significantly decreased by metfomin while expression level of E-cadherin (E-Cad) increased. Besides, metformin increased the expression levels of phosphorylated adenosine 5′-monophosphate (AMP)-activated protein kinase (p-AMPK), microtubule-associated protein (MAP) light chain 3B (LC3B) and Beclin1 proteins, and reduced levels of phosphorylated mammalian target of rapamycin (p-mTOR) and p62 proteins *in vivo* and *in vitro*. These results suggest that metformin could inhibit silica-induced pulmonary fibrosis by activating autophagy through the AMPK-mTOR pathway.

## Introduction

Silicosis is an important occupational disease and characterized by persistent lung inflammation and progressive fibrosis, which may eventually cause respiratory failure (Sayan and Mossman, 2016; Li et al., 2017). Inhaled silica particles can cause injury of lung macrophages and epithelial cells triggering an inflammatory response. Inflammation is a critical a pathogenic process of silicosis. Repeated inflammatory reactions lead to the recruitment and accumulation of inflammatory cells which secrete high levels of proinflammatory and profibrotic cytokines, such as transforming growth factor-β1 (TGF-β1), tumor necrosis factor-α (TNF-α) and interleukin-1β (IL-1β) (Fujimura, 2000; Dong and Ma, 2016). Higher level of cytokines further induces epithelial-mesenchymal transition (EMT), a process in which epithelial cells gradually lose their epithelial characteristics and acquire the mesenchymal phenotype, such as down-regulation of E-cadherin (E-Cad) and up-regulation of Vimentin. EMT is one of the important driving forces behind fibrosis through promoting the abnormal deposition of extracellular matrix (ECM) and consequent tissue remodeling and fibrotic scarring (Camara and Jarai, 2010; Stone et al., 2016; Rout-Pitt et al., 2018; Sun et al., 2019).

Autophagy is a vital regeneration process to maintain the balance of the intracellular environment through cleaning the own damaged cellular components and participating in cell proliferation and apoptosis (Mizushima et al., 2011). Autophagy has been found to play a vital role in myocardial, skin, liver, and renal fibrosis, especially in lung fibrosis (Chen S. et al., 2015). Recent studies suggested that autophagy can reduce the expression of fibrogenic factors and inhibit the deposition of collagen in fibroblasts (Hernandez-Gea et al., 2012). In addition, autophagy alleviates the silica-induced pulmonary fibrosis by decreasing apoptosis of alveolar epithelial cells in silicosis (Chen S. et al., 2015).

Autophagy and mitochondrial homeostasis are modulated by AMP-activated protein kinase (AMPK) which is a serine/threonine-protein kinase. It has been found that the AMPK signaling pathway coordinates the induction of autophagy by inhibiting mammalian target of rapamycin (mTOR) (Mihaylova and Shaw, 2011). AMPK inhibits mTORC1-dependent ULK activity by phosphorylating S317 and S777, leading to the activation of autophagy (Lawrence and Nho, 2018) and suppressing mTORC1 *via* its phosphorylation activates autophagy indirectly (Tavakol et al., 2019).

AMPK has been recognized as a cellular bioenergy sensor and metabolic regulator on the various metabolic stresses (Ha et al., 2015; Herzig and Shaw, 2018; Rangarajan et al., 2018). AMPK has been found to be a pivotal molecule that modulate the fibrogenesis by inhibiting inflammatory injury, ECM secretion, and the induction of effector cells (Jiang et al., 2017).

Drug repurposing or repositioning for different common and rare diseases is an efficient way for drug discovery because of low cost in drug development by avoiding clinical trials and de-risked compounds (Pushpakom et al., 2019). Metformin is a common biguanide antidiabetic drug for type 2 diabetes treatment. Mechanistically, metformin elicits pleiotropic effects mainly *via* activating AMPK (Tsaknis et al., 2012; Sato et al., 2016). Evidence has shown that metformin has anti-inflammatory effects and anti-fibrosis effects. Metformin has been found to be able to inhibit cardiac fibrosis induced by pressure overload *in vivo* and reduce collagen synthesis in cardiac fibrosis probably *via* inhibition of the TGF-β/Smad3 signaling pathway (Xiao et al., 2010). Moreover, metformin prevents airway remodeling in a mouse model of bronchial asthma, indicating its potential anti-fibrotic properties (Park et al., 2012). A recent study found that metformin can effectively reverse bleomycin-induced pulmonary fibrosis, suggesting that metformin has effects on idiopathic pulmonary interstitial fibrosis (Gamad et al., 2018). Since silicosis is characterized mainly by pulmonary fibrosis, we speculated that metformin may have therapeutic effects on this disease.

In this study, we explored the effects of metformin on the silicosis for which we established the rat silicosis model as well as an *in vitro* co-culture system harboring a human macrophages cells and human bronchial epithelial cells treated the with silica particles and metformin. We then investigated the pathological changes in silicosis rat and cell co-cultured model with and without metformin treatment and examined the inflammatory responses, EMT and particularly the autophagy pathways using ELISA and Western blotting. Results showed that metformin regulates autophagy through the AMPK-mTOR pathway to reduce silica particle-induced fibrosis.

## Methods and Material

### Reagents and Antibodies

Silica particles (0.5–10 μm) were purchased from Sigma Aldrich (S5631, Shanghai, China). Standard suspensions of 50 mg/ml silica particles were prepared in 0.9% normal saline and autoclaved at 120°C for 2 h. Metformin was purchased from Sino-US Shanghai Squibb Pharmaceutical Co., Ltd. (Shanghai, China), and dissolved in 0.9% physiological saline by gavage. RIPA buffer, PMSF, BCA Protein Assay kit and Ad-GFP-LC3B were purchased from Beyotime Biotechnology (C3006, Shanghai, China). Compond C was purchased from Selleck Chemicals (S7840, Houston, United States). Goat anti-rabbit IgG H&L (Alexa Fluor® 488) and Goat anti-mouse IgG H&L (Alexa Fluor® 594) and primary antibodies α-SMA (ab32575), Beclin1 (ab207612) were purchased from Abcam (Cambridge, United Kingdom), E-cadherin (14472), Vimentin (5741S), LC3B (3868), mTOR (2983S), p-mTOR (2971S), AMPK (5831S), p-AMPK (50081S) were purchased from CST (Beverly, MA, United States). IRDye 680RD Goat anti rabbit (926-68071) and IRDye 800RD Goat anti Mouse (926-32210) secondary antibodies were purchased from Li-COR (Nebraska, United States).

### *In Vivo* Experiment

#### Animals and Teatment

Forty-eight male Wistar rats (200–220 g, 6–8 weeks old) were purchased from Jinan Pengyue Experimental Animal Breeding Co., Ltd. (Jinan, China). All animals were housed under specific pathogen-free (SPF) conditions with free access to water and food. The ethical committee of Shandong Academy of Occupational Health and Occupational Medicine and the Frist Medical University approved the use of the experimental animals. The animal care and experimental protocol was approved by the ethical committee of Shandong Academy of Occupational Health and Occupational Medicine and Shandong First Medical University. Rats were randomly divided into six groups with eight rats in each, maintained under 12:12 h light-dark conditions at 23 ± 2°C and relative humidity 40–70%. Appropriate measures were taken using pain management protocol to reduce pain in animals, and the relief of pain and distress received careful attention during the experiment.

Based on reported toxicity of metformin in rats (Quaile et al., 2010) and a previous study (Gamad et al., 2018), all rats were randomly divided into six groups with eight rats in each including: negative control group, metformin control group (400 mg/kg/day), silica modal group and three metformin treatment groups (100, 200, 400 mg/kg/day). The µm-sized silica particles were prepared with normal saline as a 50 mg/ml silica suspension. The silica model group and three metformin treatment groups were injected with 1 ml (50 mg/kg) silica suspension into the lung once using a non-exposed tracheal intubation, and the rats in the negative control group and metformin control group were injected with the same volume of normal saline solution. In our previous studies, we found the treatment regime with dosage of silica particle at 50 mg/kg for 28 days yields clear manifestation of silicosis in rats (Sai et al., 2019; Pang et al., 2021). After being exposed to silica for 28 days, the rats in three metformin treatment groups were given a daily intragastric administration of 100, 200, 400 mg/kg metformin and metformin control group were given a daily intragastric administration of 400 mg/kg metformin for another 28 days. The rats in the negative control and silica model groups were treated with saline only. After treatment with metformin for 28 days, all the rats in each group were euthanized with an overdose of 150 mg/kg sodium pentobarbital (Merck and Co., Inc.) *via* intraperitoneal injection, and death in all rats was *via* observation of the cessation of respiration and palpation of the heartbeat. The lungs of each rat were harvested and used for the animal experiment (Supplementary Figure S1A).

#### Histopathological Observation

The lung tissues of rats in each group were isolated and fixed by 4% formaldehyde embedded in paraffin followed by dehydration, embedding in paraffin, and slicing onto 5 μm thick sections. Then the slides were stained with Hematoxylin and Eosin (H&E) and Masson trichrome. The pathological changes were observed under an optical microscope to examine the inflammatory infiltration, the integrity of the alveolar structure and collagen deposition under an optical microscope. The degree of alveolitis and pulmonary fibrosis was evaluated according to the scoring system outlined in Szapiel et al. (1979). Alveolitis was graded using the following criteria: None (0), no alveolitis; mild (1+), thickening of the alveolar septum by a mononuclear cell infiltrate; moderate (2+), a more widespread alveolitis; severe (3+), a diffuse alveolitis. The extent of fibrosis was graded using the following criteria: none (0), no fibrosis; mild (1+), focal regions of fibrosis, alveolar architecture has some distortion; moderate (2+), more extensive fibrosis and fibrotic still focal; severe (3+), widespread fibrosis, confluent lesions with extensive derangement of parenchymal architecture.

#### Immunohistochemistry of Lung Tissue

The lung tissue sections were deparaffinized, and antigen retrieved using citrate buffer solution. The sections were incubated with 3% H_2_O_2_ for 20 min at room temperature to eliminate endogenous peroxidase activity. After blocked with 3% BSA for 30 min, tissue sections were incubated with primary antibodies specific to E-Cad, α-SMA, Vimentin and LC3 overnight at 4°C, followed by incubation with horseradish peroxidase (HRP) labeled secondary antibody for 1 h at room temperature. After using a DAB kit for color development, the sections were counterstained with hematoxylin for 3 min, the image was observed by a fluorescence microscope (Olympus Co., Tokyo, Japan). The staining results were analyzed by Image-Pro Plus software. Integrated optical density summation (IOD SUM) of Vimentin, E-Cad, α-SMA and LC3 protein were measured by Image-Pro Plus software.

#### Enzyme-Linked Immunosorbent Assay

The homogenate samples of rat lung tissue in each group were centrifuged at 9,000 g at 4°C for 20 min. The supernatant samples were analyzed for the concentrations of TGF-β1 (ER10-96), TNF-α (ER02-96) and IL-1β (ER01-96) (Biokits Technologies Inc., Beijing, China) using rat ELISA assay kits following manufacturer’s instructions. And the total protein concentration determined were standardized using the Coomassie blue staining kit (Nanjing Institute of Biological Engineering, Nanjing, China). The absorbance was measured at 450 and 570 nm using a spectrophotometer. The results were expressed in pg/mg protein.

#### Western Blot Analysis

Total protein was extracted by homogenization in ice-cold RIPA buffer with 1 mM PMSF. Homogenates were centrifuged for 15 min at 12,000 g. Protein concentrations in the supernatants were calculated using a BCA Protein Assay kit. The protein extracted was separated by SDS-PAGE and then electrotransferred to PVDF membrane (Merck KGaA, Darmstadt, Germany). The membranes were initially blocked with 5% non-fat dry milk in phosphate-buffered solution (PBS) for 1 h, and subsequently incubated with primary antibody against, including α-SMA (1:1,000), E-Cad (1:1,000), Vimentin (1:1,000), Beclin1(1:1,000), LC3B (1:1,000), mTOR (1:1,000), p-mTOR (1:1,000), AMPK (1:1,000), p-AMPK (1:1,000) incubated overnight at 4°C. Blots were washed for three times with Tris-Buffered Saline and 0.1% Tween 20 (TBST) and followed by IRDye 680RD (1:5,000) and IRDye 800RD (1:5,000) secondary antibodies for 1 h at room temperature. Finally, the densities were scanned by Li-Cor and quantified using the Image Studio Software.

### *In Vitro* Experiment

#### Cell Culture

The bronchial epithelial cell line HBEC was presented by the Centre for Clinical Research of Queensland, Australia. And the THP-1 cell line was obtained from the American Type Culture Collection (ATCC) (Manassas, VA, United States), and were maintained at 37°C with 5% CO_2_. The HBEC was cultured in PneumaCult-ExPlus Medium (05401, Stemcell Technologies, Canada) supplemented 1% Antibiotic-Antimycotic (Gibco, United States) and 0.1% Hydrocortisone Stock Solution (Stemcell Technologies, Canada). The THP-1 cell was cultured in RPMI 1640 (Gibco, United States) supplemented 10% FBS (BI, Israel) and 1% Antibiotic-Antimycotic.

#### Cell Counting Kit-8 Assay

4 × 10^3^/well HBEC were seeded in 96-well plates and incubated overnight, and the medium was changed to the presence of various doses of metformin (0, 0.1, 0.25, 0.5, 1, 2, 5, 10 mM) at various time points (24, 48, and 72 h). The selection of treatment concentrations was based previous studies (Wang et al., 2015; Mishra and Dingli, 2019). Cell viability was measured using the CCK-8 assay kits (CK04, Dojindo Laboratories, Kyushu, Japan) in accordance with the manufacturer’s instructions. The absorbance at 450 nm was measured using a microplate reader.

#### THP-1 Differentiation and Co-Culture

THP-1 cells (1 × 10^6^/well) were plated in transwell inserts (Corning, Lowell, United States) with a membrane pore size of 4 μm and were treated with 100 ng/ml phorbol 12-myristate 13-acetate (PMA, Sigma, MO, United States) for 72 h to differentiate into macrophage-like forms. HBEC (4 × 10^4^/well) were cultured alone at the bottom of 6-well plate for 2 days. Then, the cell culture inserts containing THP-1 macrophages were transferred to the plates containing HBEC. To confirm if the metformin regulates the EMT process of HBECs by AMPK-dependent activation of autophagy, we added metformin and compound C (CC) an AMPK Inhibitor. The experiment was divided into six groups: control group (Control), metformin control group (Met), silica group (Silica), silica and metformin intervention group (Silica + Met), silica and CC intervention group (Silica + CC) and silica, metformin and CC intervention group (Silica + Met + CC).

With macrophages differentiated from THP-1 cells already seeded, 100 μg/ml of silica solution was introduced into the insert in the silica particle group, silica and metformin group, silica and CC group and silica, metformin and compound C group. Meanwhile, in the metformin control group and silica and metformin intervention group, 0.5 mM metformin solution was added to the bottom, silica and CC group was added 1 μM CC, metformin and CC group was added 0.5 mM metformin and 1 μM CC. The control group had no intervention. Each sample was cultured in duplicate, and each co-culture experiment was repeated 3 times (Supplementary Figure S1B).

#### Immunofluorescence Staining

Cell slides were placed in the culture chamber. After 72 h of co-culture treatment, the HBEC were washed three times with phosphate-buffered saline (PBS), fixed with 4% paraformaldehyde, and then blocked with blocking buffer (P0260, Beyotime biotechnology, Shanghai, China) for 1 h at room temperature. The cells were then incubated with E-Cad, Vimentin and α-SMA at a dilution of 1:200 overnight at 4°C, followed by incubation with secondary antibodies (1:500) for 1 h at room temperature. 4′,6-diamidino-2-phenylindole (DAPI) was used for nuclear staining. Finally, cells were observed and photographed by a fluorescence microscope (Olympus, Tokyo, Japan). The mean fluorescence was detected by the ImageJ software.

#### Enzyme-Linked Immunosorbent Assay

After receiving the indicated interventions for 72 h, the levels of TGF-β1 (EH03-96), TNF-α (EH02-96) and IL-1β (EH18-96, Biokits Technologies Inc., Beijing, China) in the cell supernatant of HBEC in each group were determined using ELISA as per the manufacturer’s instructions. Then the total protein concentration was determined and standardized using the Coomassie blue staining kit (Nanjing Institute of Biological Engineering, Nanjing, China). The absorbance was measured at 450 and 570 nm using a spectrophotometer. The results were expressed in pg/mg protein.

#### Ad-GFP-LC3 Transfection

HBECs (4 × 10^4^/well) were cultured alone at the bottom of 6-well plate for 24 h. After a wash with fresh culture medium, cells were transfected with Ad-GFP-LC3 adenovirus at a MOI of 100 in 2 ml culture medium for 48 h at 37°C. And after 72 h intervention by co-culture system, HBEC cells were observed and photographed by a fluorescence microscope.

#### Western Blotting Analysis

After the cell co-culturing, HBEC were washed with cold phosphate-buffered saline (PBS). The proteins were extracted using RIPA buffer with 1 mM PMSF. Protein concentrations were calculated using a BCA Protein Assay kit. Samples containing equivalent amounts of lysate protein (30 μg) were separated on SDS-PAGE and transferred to a PVDF membrane. Western Blotting was used to detect the effect of the co-culture model of silicosis on the expression of EMT-related proteins (E-Cad, Vimentin, α-SMA), autophagy-related proteins (LC3B, Beclin1, p62) and AMPK, p-AMPK, mTOR and p-mTOR.

### Statistical Analysis

Statistical analyses were performed using SPSS 22.0 software (IBM Corp.). All data are shown as the mean ± SD. And all data were checked for normality and homoscedasticity, differences between groups were performed using one-way ANOVA followed by LSD-test, while Dunnett’s method was used for variance differences. Non-parametric data was represented by median and to analyze the data using Kruskal-Wallis analysis of variance. When statistical significance is obtained, the rank-based Mann–Whitney *U*-test be used to compare the groups. The *p* values < 0.05 were regarded as statistically significant.

## Results

### Metformin Moderates the Effects of Silica Exposure on Body Weight and Lung Organ Coefficient

**FIGURE 1:**
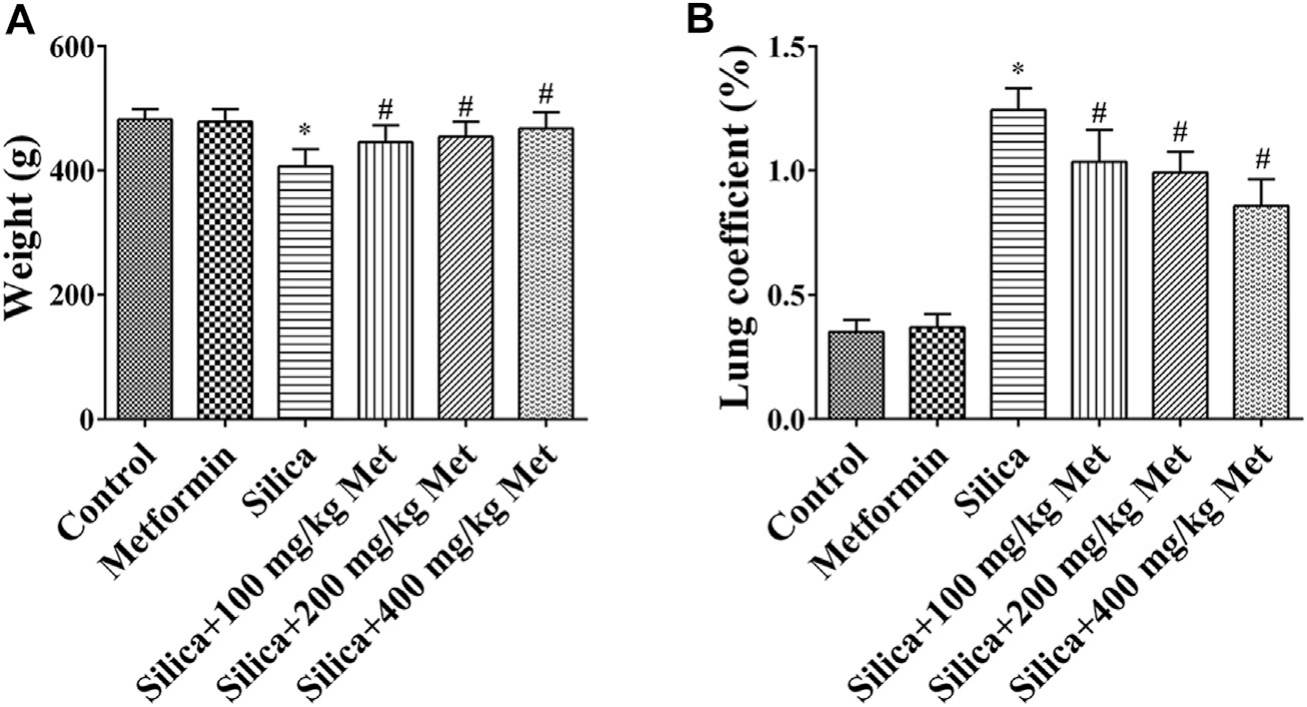
Metformin moderates the effects of silica exposure on rat body weight and lung organ coefficient. The **(A)** body weight and **(B)** lung coefficient of the rats exposed to silica particles considerably reduced (about 15%) and significantly increased, respectively, when compared with that in rats from control group. Metformin treatment recovered the body weight and lung coefficient with significant changes. All the data are presented as mean ± SD (*n* = 8 for each group). *p < 0.05, compared to the control group; ^#^ *p* < 0.05, compared to silica group.

The weight of the rats after exposed to silica particles was considerably reduced (about 15%) when compared with that in rats from control group. Compared with the silica group, the weight of animals in the metformin treatment groups increased significantly (*p* < 0.05). The lung organ coefficient of the silica group was significantly higher than the control group, while the lung organ coefficient was significantly reduced after metformin treatment (*p* < 0.05) (Figure 1).

### Metformin Effectively Alleviates Pulmonary Inflammation and Fibrosis Mediated by Silicon Dioxide in Rats

**FIGURE 2:**
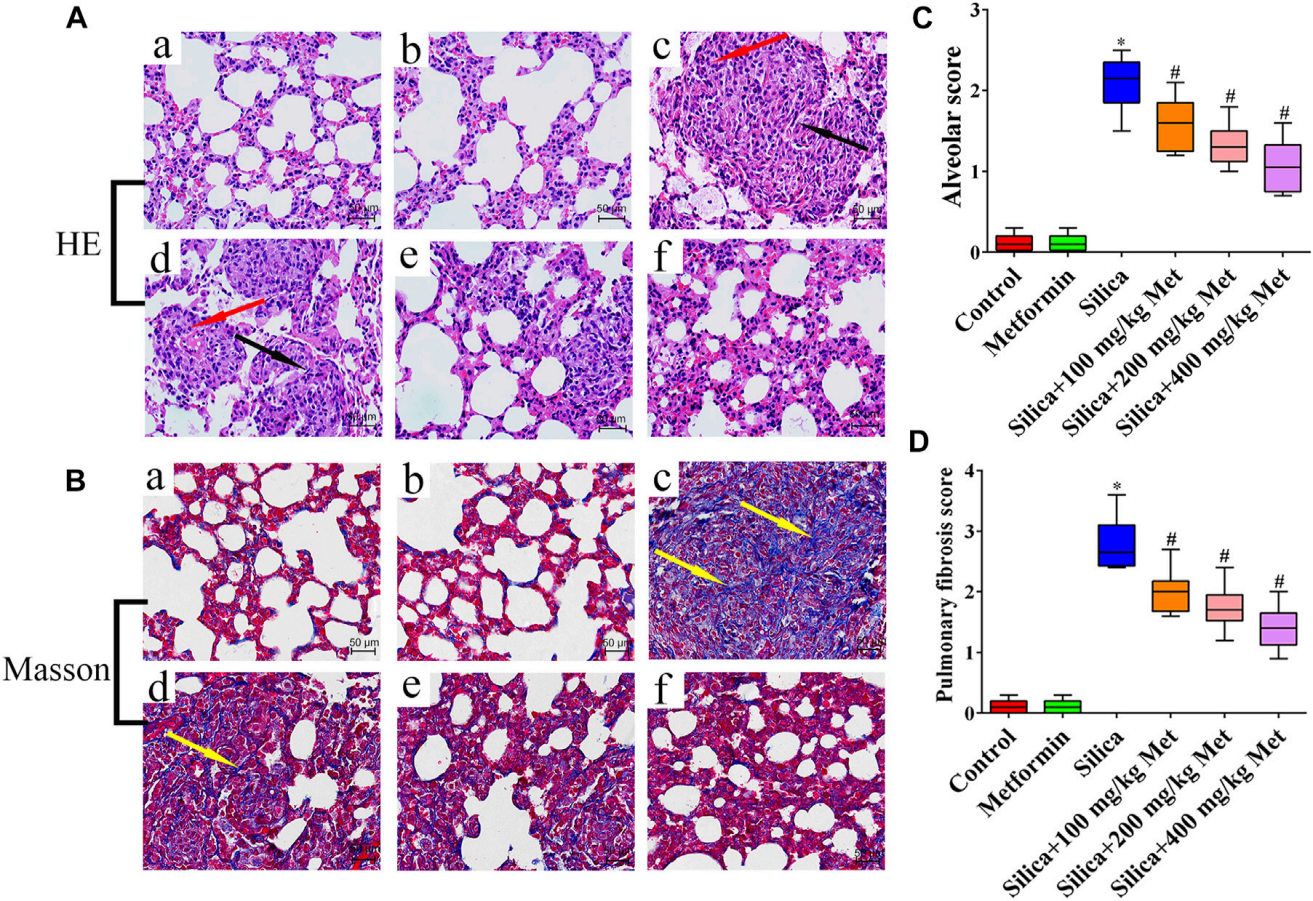
Metformin reduces the inflammation and collagen accumulation in the lung tissue of silicosis rat caused by silica. **(A)** HE staining of lung tissues (200 × mag.) The red arrows point to the inflammatory cells. The fibroblasts are labeled with black arrows. **(B)** Masson trichrome staining of collagen on lung sections (200 × mag.). The yellow arrow points to the collagen. **(C-D)** Quantitative analysis of rat alveolitis and fibrosis score. **a** Control group; **b** Metformin treatment group; **c** Silica group; **d** Silica+100 mg/kg metformin group; **e** Silica+200 mg/kg metformin group; **f** Silica+400 mg/kg metformin group. *p < 0.05, compared to the control group; ^#^ *p* < 0.05, compared to silica group.

HE staining results showed that the rats in the negative control group and the metformin control group have an intact lung structure, with normal alveolar septa and no obvious inflammatory changes (Figure 2A: a-b). However, the lung tissues of the rats from silica group (Figure 2A: c) showed a severe inflammatory response indicated by the thickness of alveolar septal increased considerably, neutrophils infiltration and monocytes around the alveolar stroma, mainly macrophages. In contrast, after metformin treatment for 28 days, alveolitis was significantly reduced, the alveolar structure was significantly improved, and alveolar inflammation was also significantly relieved (Figure 2A: d-f). Using the Szapiel (Szapiel et al., 1979) method, we quantified alveolar inflammation and the results showed that the alveolar score of the silica group was almost 2 times higher than the rats from negative control group. However, after metformin treatment, even at the concentration of 100 mg/kg, the alveolar inflammation score was about 25% lower than in the silica group (*p* < 0.05). The inflammation scores were decreased in a dose-repose pattern (Figure 2C). Masson staining indicates the degree of pulmonary fibrosis by staining collagen fibers. Collagen deposition (blue areas) in the lungs of rats from the silica group was significantly increased, compared with the control and metformin group (Figure 2B: c). However, with metformin treatment, inflammatory cells and the accumulation of collagen fibers were significantly reduced (Figure 2B: d-f). Quantitative analysis showed that the pulmonary fibrosis score of the silica group was significantly higher than the negative control group. However, the pulmonary fibrosis scores decreased from 2.8 of the silica group to 2, 1.8, and 1.5 after treatment with metformin at 100, 200, and 400 mg/kg (*p* < 0.05). (Figure 2D).

### Metformin Reduces Silica Particle-Induced Inflammation by Inhibiting Inflammatory Cytokines TGF-β1, TNF-α, and IL-1β in Lung Tissues

**FIGURE 3:**
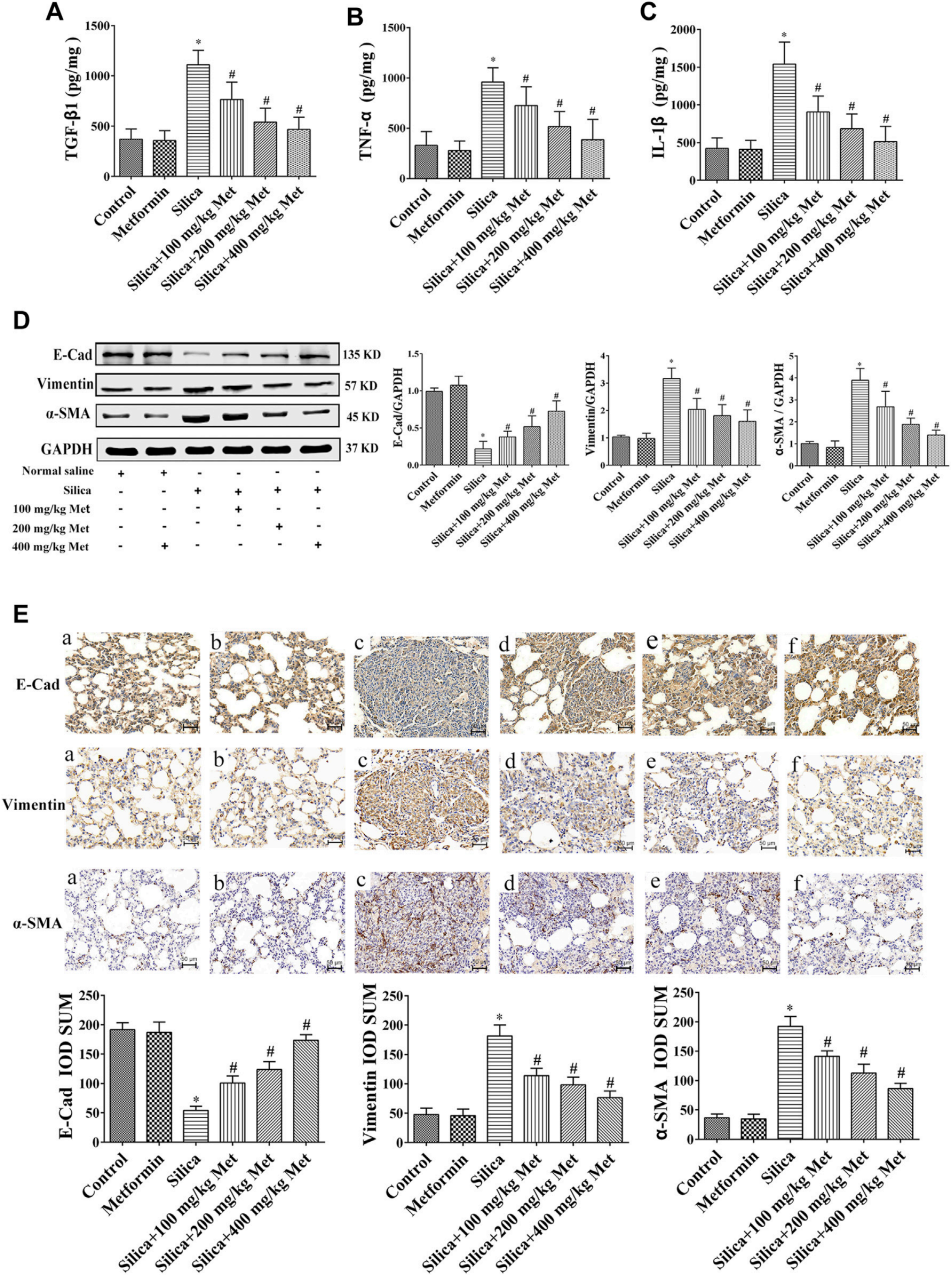
Metformin reduces the expression level of inflammatory factors and alleviates silicosis fibrosis induced by silica particles in rat lung tissue. The expression level of **(A)** TGF-β1, **(B)** TNF-α and **(C)** IL-1β detected by ELISA. **(D)** The expressions of EMT-associated proteins expression level in the lung tissue of rats were detected by Western blot and **(E)** Immunohistochemistry (200 × mag.). **a** Control group; **b** Metformin treatment group; **c** Silica group; **d** Silica+100 mg/kg metformin group; **e** Silica + 200 mg/kg metformin group; **f** Silica + 400 mg/kg metformin group. All the data are presented as mean ± SD (*n* = 8 for each group). *p < 0.05, compared to the negative control group; ^#^ *p* < 0.05, compared to silica group. TGF-β1, transforming growth factor-β1; TNF-α, tumor necrosis factor-α; IL-1β, interleukin-1β; Pro, protein; EMT, epithelial-mesenchymal transition; E-cad, E-Cadherin; α-SMA, α-Smooth muscle actin; Met, metformin.

As shown in Figures 3A-C, TGF-β1, TNF-α and IL-1β in lung tissues of rats from control and metformin group showed a basal level less than 500 pg/mg protein. Exposure to silica particles caused significantly increase of these cytokines on the 56th days (*p* < 0.05). However, metformin treatment led to a dose-response reduction of the inflammatory cytokines (*p* < 0.05). Metformin treatment at 100 mg/kg led to about 30, 20, and 40% reduction of TGF-β1, TNF-α, and IL-1β, respectively. Higher concentration of metformin (400 mg/kg) caused over 50% reduction of these cytokines. This indicates that metformin inhibits the expression of inflammatory cytokines in rat lung tissue caused by silica, thereby reducing alveolar inflammation.

### Metformin Reversed Silica-Induced EMT in the Lung of Rats

E-Cad, α-SMA and Vimentin which are hallmark proteins for EMT were measured by the Western Blot and Immunohistochemistry. Rats with Metformin treatment only showed similar level of these proteins in lung tissues as that in control group. In the lung tissue of rats from the silica group, the expression level of E-Cad was reduced about three quarters while α-SMA and Vimentin were increased about 3 and 4 times, respectively, compared with the rats from control group. However, in silicosis rats treated with metformin, the expression level of E-Cad but α-SMA and Vimentin were significantly recovered in a dose-response relation with the concentration of metformin compared with the silica group (*p* < 0.05) (Figures 3D,E). These suggested that metformin could inhibit the process of EMT.

### Metformin Inhibits Pulmonary Fibrosis by Activating Autophagy *via* the AMPK-mTOR Signaling Pathway

Recent studies have suggested the contribution of autophagy to the benefit of metformin. Therefore, we explored the involvement of autophagy in the effects of metformin on silicosis by assessing the expression of p62, Beclin 1 and LC3. The results showed silica dust treatment almost 30% decrease of p62 and, 30% increase of Beclin1 and LC3, respectively, in comparison with rats from control group. Treatment of metformin led to further significant reduction of p62 and increase of Beclin1 and LC3 (*p* < 0.05). These results indicate that silica exposure could induce autophagy which was promoted further by metformin treatment.

**FIGURE 4:**
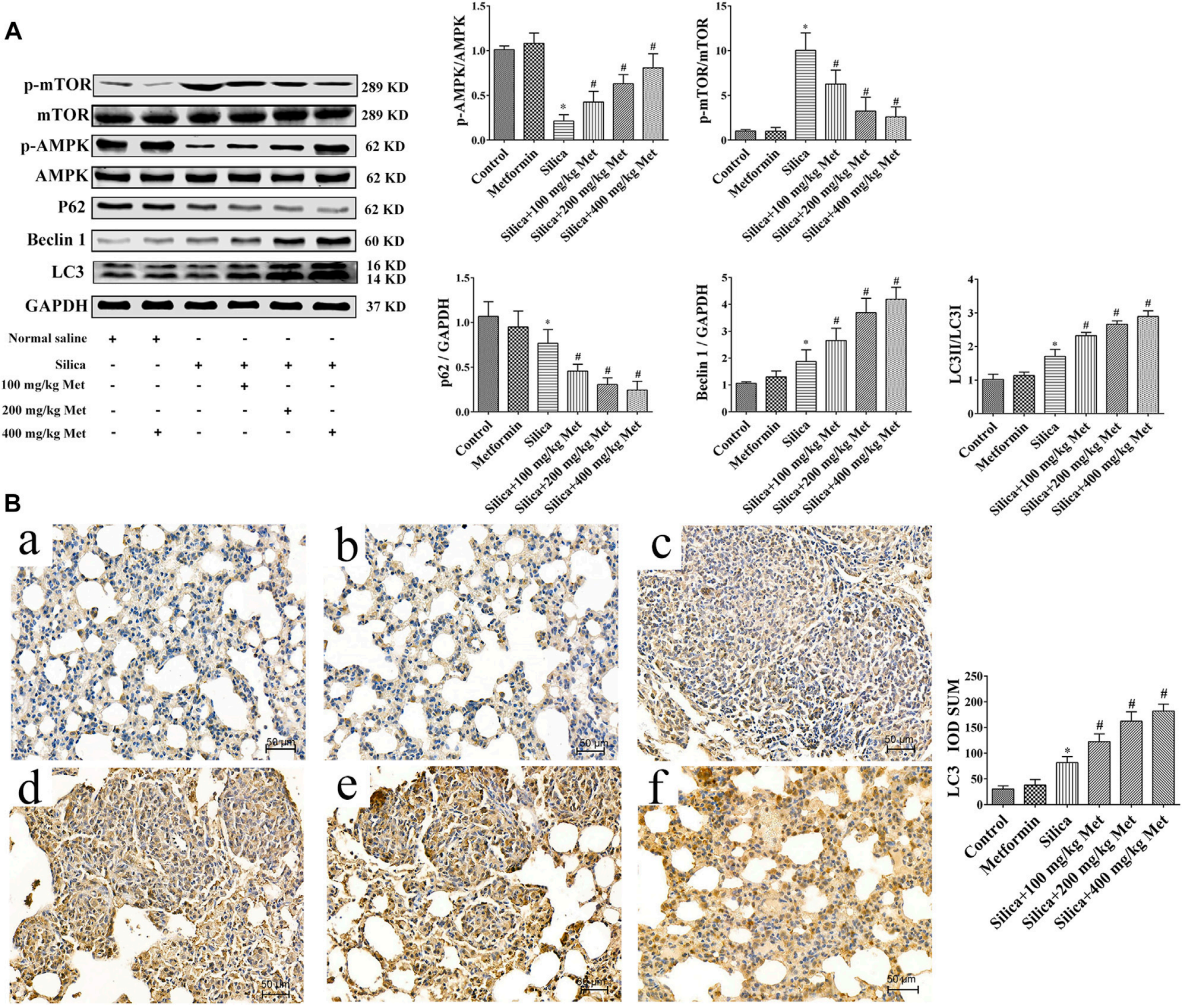
Metformin activates autophagy *via* the AMPK-mTOR signaling pathway. **(A)** The protein levels of AMPK, p-AMPK, mTOR and p-mTOR, p62, Beclin1, and LC3 in lung tissues of rats by western blot. **(B)** The protein levels of LC3 was determined by Immunohistochemistry (200 × mag.). **a** Control group; **b** Metformin treatment group; **c** Silica group; **d** Silica+100 mg/kg metformin group; **e** Silica+200 mg/kg metformin group; **f** Silica+400 mg/kg metformin group. All the data are presented as mean ± SD (n = 8 for each group). *p < 0.05, compared to the negative control group; ^#^ *p* < 0.05, compared to silica group. AMPK, adenosine5′-monophosphate (AMP)-activated protein kinase; mTOR, mammalian Target of Rapamyc; LC3, microtubules associated protein 1 light chain 3; Met, metformin.

And we then examined the p-AMPK, AMPK and p-mTOR, mTOR by WB. The results showed that there was no difference between the rats in control and metformin only group in the protein level of p-AMPK and p-mTOR. Rats from silica dust group showed lower level of p-AMPK but higher in p-mTOR compared with rats from the control and metformin only group (*p* < 0.05). However, metformin treatment resulted in one time increase of the expression level of p-AMPK. And meanwhile p-mTOR was obviously decreased in a dose-response manner compared with the silica group (*p* < 0.05) (Figure 4A).

### Metformin Inhibit EMT Process *in vitro*

**FIGURE 5:**
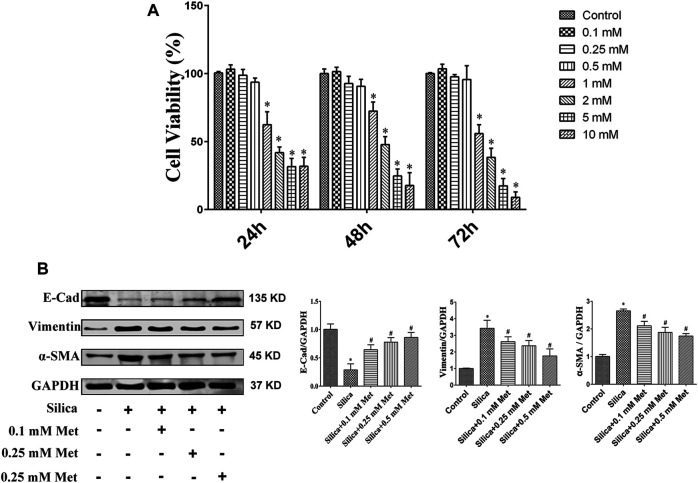
Different concentrations of metformin affect cell viability and inhibit EMT process. **(A)** The viability of HBEC incubated with different concentrations of metformin was detected by the CCK-8 assay. **(B)** Western blotting results for the expressions level of EMT-associated proteins on HBEC after co-culture with different concentrations of metformin. All the data are presented as mean ± SD (*n* = 3 for each experimental group). *p < 0.05, compared to the control group; ^#^ *p* < 0.05, compared to silica group. EMT, epithelial-mesenchymal transition; E-cad, E-Cadherin; α-SMA, α-Smooth muscle actin; Met, metformin.

To identify suitable dosage levels, we first tested the cytotoxicity of metformin on HBECs with different concentrations of metformin (0, 0.1, 0.25, 0.5, 1, 2, 5, 10 mM) for 24, 48, and 72 h. The dosage selection was referred to previous studies (Wang et al., 2015; Guo et al., 2016), in which the cytotoxicity of metformin was tested at the concentration 0–50 mM. As shown in Figure 5A, metformin at 0.1–0.5 mM had no significant effect on cell growth at all time intervals. Compared with control group, the viability of HBEC incubated with 1, 2, 5, and 10 mM of metformin were both significantly reduced and less than 85% on the 24, 48 and 72 h (*p* < 0.05). Therefore, 0.1, 0.25, and 0.5 mM of metformin were used for following *in vitro* experiments. After THP-1 cells differentiated into macrophages, 100 μg/ml of silica solution was introduced into the insert in the silica group and metformin group. Meanwhile 0.1, 0.25 and 0.5 mM metformin was added to the bottom in metformin group. The cells were cultured for 72 h. E-Cad, α-SMA and Vimentin were measured to assess the effect of metformin on EMT. The WB results showed silica caused an over 70% decrease of E-Cad which was recovered significantly by treatment of metformin. Compared with control cells, the expression of α-SMA and Vimentin was increased more than 2 times by silica treatment. However, co-treatment of metformin at 0.1, 0.25 and 0.5 mM led to a significant decrease of these two proteins in a dose-response manner (Figure 5B).

### Metformin Reduces Silica Particle-Induced Inflammation in Human Bronchial Epithelial Cells by Inhibiting Inflammatory Cytokines TGF-β1, TNF-α, and IL-1β

**FIGURE 6:**
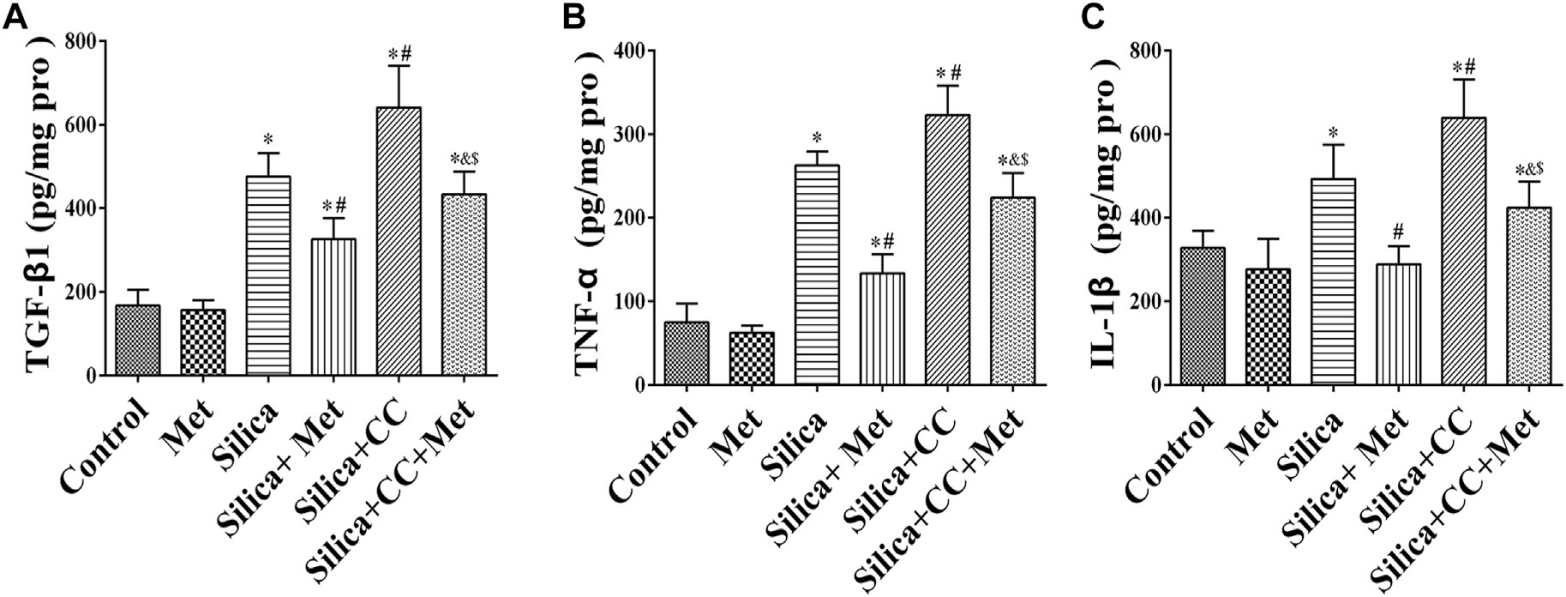
Metformin reduces the expression level of inflammatory factors by silica particles in HBECs. The expression level of **(A)** TGF-β1, **(B)** TNF-α, and **(C)** IL-1β detected by ELISA. All the data are presented as mean ± SD (*n* = 3 for each experimental group). *p < 0.05, compared to the negative control group; ^#^ *p* < 0.05, compared to silica group; ^&^ *p* < 0.05, compared to Silica + Met group; ^$^ *p* < 0.05, compared to Silica + CC group. TGF-β1, transforming growth factor-β1; TNF-α, tumor necrosis factor-α; IL-1β, interleukin-1β; Pro, protein; Met, metformin; CC, Compound C.

The content of TGF-β1, TNF-α, and IL-1β in HBECs of the co-culture system was measured using ELISA. The levels of TGF-β1, TNF-α, and IL-1β in the silica group were significantly higher in silica treatment compared with the control group (*p* < 0.05). silica group with metformin treatment led to a 30, 50, and 40% reduction of TGF-β1, TNF-α, and IL-1β, respectively compared with that in the silica group (*p* < 0.05). To explore the role of AMPK in the effect of metformin, we measured the level of cytokines in cells treated with silica and CC, or silica, metformin and CC. The results showed co-treatment of silica and CC led to a 30% increase of these cytokines with the significant difference in comparison with that in silica group (*p* < 0.05). The increased level of cytokines was reduced significantly by treatment with metformin (*p* < 0.05) as shown in Figure 6.

### Metformin Regulates the Expression of EMT-Related Proteins in Human Bronchial Epithelial Cells of Co-Culture System Exposed to Silica Particles

**FIGURE 7:**
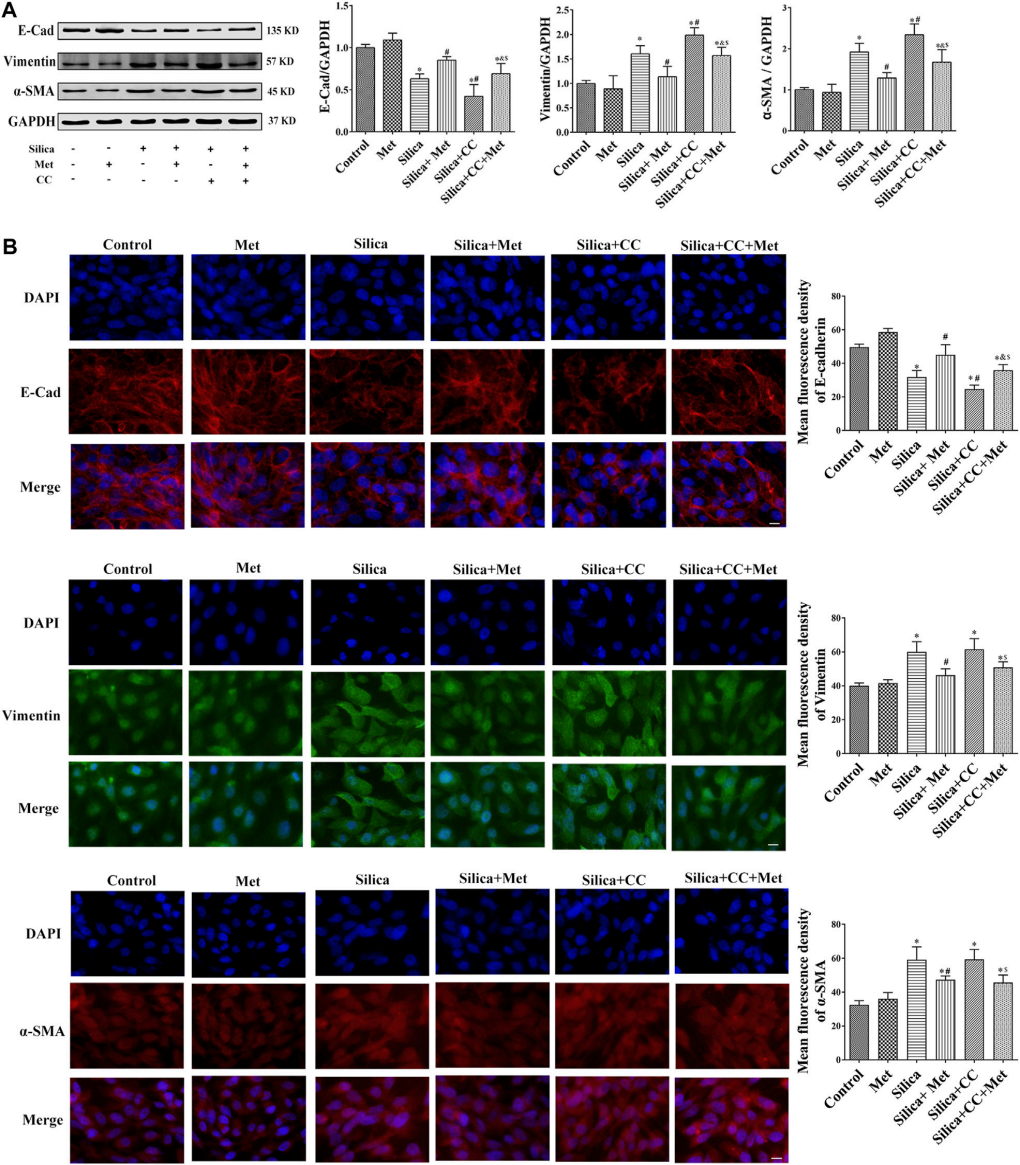
Metformin regulates the expression of EMT-related proteins induced by silica particles. **(A)** The expression level of the expressions of EMT-associated proteins expression level in HBEC cells were detected by Western blot and the E-Cad, Vimentin and α-SMA were detected by **(B)** Immunofluorescence. Scale bar, 10 μm. All the data are presented as mean ± SD (*n* = 3 for each experimental group). *p < 0.05, compared to the negative control group; ^#^ *p* < 0.05, compared to silica group; ^&^ *p* < 0.05, compared to Silica + Met group; ^$^ *p* < 0.05, compared to Silica + CC group. EMT, epithelial-mesenchymal transition; E-cad, E-Cadherin; α-SMA, α-Smooth muscle actin; Met, metformin; CC, Compound C.

To further explore the role of AMPK in silica particle-induced EMT and the regulation of metformin, we examined the EMT proteins in cells treated with silica particles, metformin and CC using Western Blot and Immunofluorescence. The results showed that in the silica group, the expression level of epithelial marker E-Cad in HBECs was reduced 30% and co-treatment of silica with CC reduced 50% of that in control cells. Treatment with CC promoted about 20% of silica-induced Vimentin and α-SMA, which was reduced to the similar degree by metformin with significant difference (*p* < 0.05) when compared each other as shown in Figures 7A,B.

### Metformin Activates AMPK-mTOR Signaling Pathway to Up-Regulate Autophagy-Related Proteins in the Silicosis Co-culture Model

**FIGURE 8:**
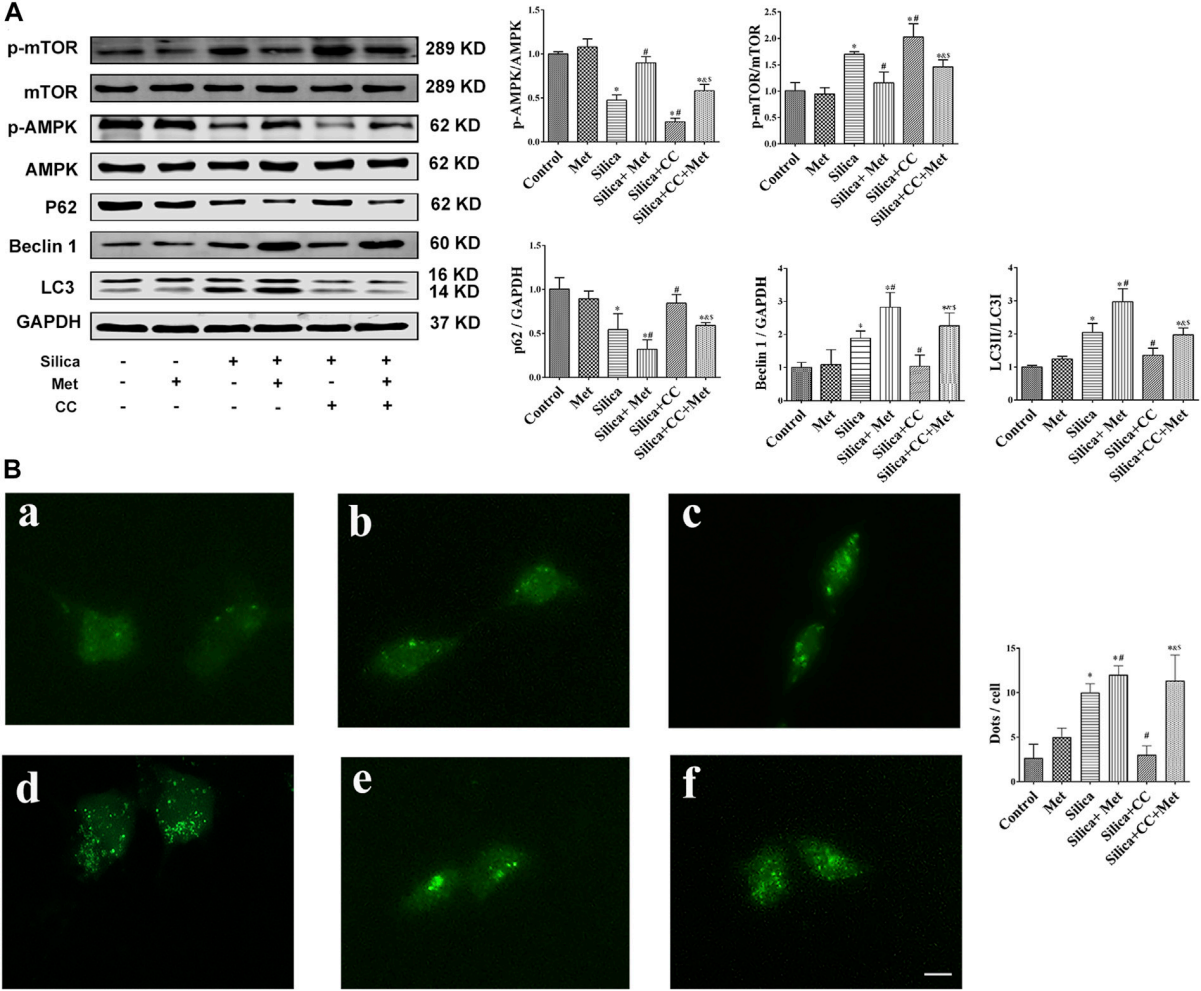
Metformin activates autophagy *via* the AMPK-mTOR signaling pathway. **(A)** The protein levels of AMPK, p-AMPK, mTOR and p-mTOR, p62, Beclin1 and LC3 in HBEC were detected by western blot. **(B)** Fluorescence images of Ad-GFP-LC3 in HBEC cells after co-culture 72 h. GFP fluorescence indicated by green puncta. **a** Control group; **b** Met treatment group; **c** Silica group; **d** Silica+0.5 mM Met group; **e** Silica+0.1 μM CC group; **f** Silica+0.5 mM Met +0.1 μM CC group. Scale bars, 30 μm. All the data are presented as mean ± SD (*n* = 3 for each experimental group). *p < 0.05, compared to the negative control group; ^#^ *p* < 0.05, compared to silica group; ^&^ *p* < 0.05, compared to Silica + Met group; ^$^ *p* < 0.05, compared to Silica + CC group. AMPK, adenosine5′-monophosphate (AMP)-activated protein kinase; mTOR, mammalian Target of Rapamyc; LC3, microtubules associated protein 1 light chain 3; Met, metformin; CC, Compound C.

To confirm if the regulation of metformin on the silica particle-induced EMT process is through AMPK-dependent activation of autophagy, we added metformin and CC, and examined the autophagy-related proteins p62, Beclin1 and LC3 by WB first. The results showed there is not much change of these proteins in HBECs of the co-culture system from control and metformin groups. Treatment with silica only led to a 30% decrease of p62 and 40% increases of Beclin1 and LC3, which was further reduced 20% for p62 and increased 25% for Beclin1 and LC3, by co-treatment with metformin. Cells co-treated with silica and CC showed higher level of p62 than that of silica group but similar to that in control cells. However, co-treatment with metformin, silica and CC led to a 25% decrease of p62 but increase in Beclin1 (50% increase) and LC3 (25% increase) in comparison with that in group of silica and CC as shown in Figure 8A.

Furthermore, we assessed autophagy by examining the formation of autophagosomes using HBECs transfected with GFP-LC3 adenovirus. As shown in Figure 8B-a and b, cells of control and metformin treatment only showed basal level of GFP-LC 3 foci. Cells treated with silica particles showed higher number of foci. (Figure 8B: c). As shown in Figure 8B: d, much more foci were found in cells treated with silica and metformin. In addition, metformin enhanced LC foci in the cells treated with silica and CC.

Then, we measured the expression of p-AMPK, AMPK, p-mTOR and mTOR using WB. The results showed that the p-AMPK expression was decreased about 50%, while the p-mTOR expression was increased about 40% of control cells in the silica group, with statistically significant differences compared with the control group (*p* < 0.05). Treatment with CC with silica particles exacerbated the change of p-AMPK and p-mTOR expression with significant difference (*p* < 0.05). However, co-treatment Metformin with silica or silica and CC significantly recovered the level of these proteins.

## Discussion

Silicosis is one of the important occupational respiratory diseases caused by inhalation of respirable crystalline silica (Fernandez Alvarez et al., 2015). As one of the most common occupational disease, silicosis occurs not only in developing countries because of poor protection facility and regulations, but also in developed countries in recent years among the stone masons (Rees and Murray, 2007). Although great efforts have been made in prevention and treatment, there has been no effective therapeutic drugs so far and lung transplantation has been still the effective option for curing silicosis.

Silicosis is mainly caused by the deposition of silica particles in the alveoli. Accumulated silica particles in the lung alveoli stimulate lung microphages and epithelial cells leading to repeated inflammation and high expression of inflammatory cytokines such as TNF-α and IL-1, which then cause fibroblasts producing collagen, leading to fibrosis and silicon nodules (Pollard, 2016). For *in vivo* study, we set up the animal silicosis model by exposing rats to silica particles according to the method in our previous work (Sai et al., 2019). Firstly, we examined the effects of metformin on body weight of the rats treated with silica. Our results showed that after being exposed to silica for 56 days, the weight of the rats exposed to silica particles reduced about 15% compared with the rats of control group (Figure 1A), and the lung coefficient was significantly increased (Figure 1B). HE staining results showed that the lung tissues of the rats from silica group exhibited severe inflammation indicated by the destroyed alveolar structure and a large number of infiltrating inflammatory cells (Figure 2A). Masson staining of lung tissue sections showed that a large amount of collagen deposition and occurrence of silicotic nodule, indicating pulmonary fibrosis occurred after exposure to silica particles (Figure 2B). In addition, in order to simulate the process of silicosis, a modified co-culture cell model *in vitro* was established according to our published study (Pang et al., 2021). Treatment with silica caused an increased level of pro-inflammatory factors including TGF-β1, TNF-α and IL-1β in lung tissue of rats and medium of the co-culture system. These results indicted an obvious inflammatory response induced by silica particles (Figures 3A–C, Figures 6A–C).

Although the mechanism of silicosis fibrosis remains to be elusive, EMT is a recognized as a critical process leading to fibrotic changes. EMT plays an important role in many lung diseases such as chronic obstructive pulmonary disease and pulmonary fibrosis (Jolly et al., 2018). During EMT, epithelial cells undergo morphological changes including cell-cell adhesions loosed, epithelial markers down-regulated, mesenchymal markers up-regulated, and an elongated fibroblast-like morphology acquired (Gabasa et al., 2017; Li et al., 2018). EMT is complex process mediated by several key transcription factors and finely regulated through epigenetic and post-translational modifications (Serrano-Gomez et al., 2016). Despite the complex and transient nature of EMT, several hallmarks have been identified for EMT assessment including epithelial markers such as E-Cad and mesenchymal markers including Vimentin and α-SMA which were investigated in this study. The results showed that the expression of E-Cad decreased and mesenchymal markers Vimentin and α-SMA increased in rat lung tissues and HBEC cells exposed to silica (Figure 3D, Figure 7).

The inhaled silica particles are engulfed by alveolar macrophages and subsequently lead to the death of alveolar macrophages during which intracellular silica, cytotoxic oxidants and inflammatory cytokines are released (Davis et al., 1996; Yang et al., 2016; Barohn et al., 2019). These factors promote the proliferation of lung fibroblasts, the production of collagen and eventually lead to the formation of fibrosis (Guo et al., 2019). Autophagy may play an important role in the lung fibrosis (Racanelli et al., 2018), but the underlying mechanisms remain elusive. Autophagy is a fundamental intracellular catabolic process for recycling damaged organelles and proteins *via* the lysosome-mediated degradation pathway (Zhao et al., 2020). This process is essential for maintaining cellular homeostasis (Tseng et al., 2019). Beclin 1 is a mammalian homolog of yeast Atg6, which is the first mammalian autophagy protein to be described (Liang et al., 1998). Free Beclin 1 is an initiator of autophagy and thus extensively used as a marker for monitoring the onset of autophagy (Cao and Klionsky, 2007). Therefore, as an important regulator of autophagy, the expression level of Beclin 1 represents autophagy activity to some extent (Xu G. et al., 2019). LC3 is a mammalian homolog of yeast Atg8 and has LC3-I and LC3-II two subforms. The conversion of LC3-I into LC3-II is a key step in autophagosome formation (Mizushima et al., 2010). Therefore, the ration of LC3-II/LC3-I is commomly used to assess the autophagy activity. p62 has been known as one of the selective substrates for LC3. When autophagy occurs, p62 first binds to the ubiquitinated protein and then combines with LC3-II localized on the inner membrane of the autophagic vacuole to form a complex (Komatsu and Ichimura, 2010). In this study, we analyzed the expression of the autophagy-associated protein LC3-II/LC3-I, Beclin1 and p62 in lung tissues of the rats and HBEC cells. Our results showed that LC3 and Beclin1 in rats and HBECs were significantly increased and p62 decreased after being exposed to silica (Figure 4, Figure 8A). This result clearly indicated silica-induced autophagy activity. Supportively, Jessop et al. (2016) showed that silica exposure causes increased expression of LC3-II *in vitro* and enhanced autophagic activity in alveolar macrophages isolated from silica-exposed mice. Chen et al. (2013) found that silica dust exposure can induce autophagy in the lung tissue of rats. Duan et al. (2014) demonstrated that Nano-SiO_2_ could induce inflammatory response, activate autophagy, and eventually lead to endothelial dysfunction. Previous studies indicated that autophagy in the macrophages can be activated by silica, characterized as the accumulation of autophagosomes, which may be associated with the silicosis progression (Chen S. et al., 2015; Liu et al., 2016). (Cheng et al., 2019) found that SiO_2_ induces activation of autophagy in human pulmonary fibroblasts cells. Recently, the activation of autophagy, a lysosome-dependent cell degradation pathway, by silica nanoparticles has been identified in alveolar epithelial cells (AECs) (Zhao et al., 2019). Additionally, Li et al. (2021) demonstrated that SiO_2_ exposure can induce pulmonary fibrosis along with autophagy both *in vivo* and *in vitro*, and autophagy might play a protective role in the progression of pulmonary fibrosis.

AMPK is a key energy sensor and regulates cellular metabolism to maintain energy homeostasis and restore energy balance at the cellular and physiological levels during metabolic stress (Garcia and Shaw, 2017). mTOR is one of the downstream targets of AMPK, and activation of AMPK can result in inhibition of mTOR signaling (Xu et al., 2012). Studies had shown that AMPK activation could inhibit TNF-α, IL-1β, and IL-6 synthesis in macrophage (Sag et al., 2008; Yang et al., 2010; Galic et al., 2011). AMPK exerts a significant anti-inflammatory effect *via* suppression of the NF-κB signaling pathway (Salminen et al., 2011). In addition, emerging evidence indicated that AMPK plays an important role in autophagy (Bujak et al., 2015; Tamargo-Gomez and Marino, 2018) through the AMPK/mTOR signaling pathway (Kim et al., 2011; Wang et al., 2019). Recent studies found that AMPK functions are strongly associated with fibrogenesis (Jiang et al., 2017). Increasing evidence has revealed that AMPK protects against fibrosis in the heart (Zhang et al., 2011), liver (Yang et al., 2015), lung (King et al., 2012), kidney (Cavaglieri et al., 2015), and skin (Takata et al., 2014). In this respect, loss or reduction of AMPK has been implicated in diabetes mellitus, obesity and aging (Burkewitz et al., 2014; Southern et al., 2017; Rana et al., 2020). To explore the possible role of AMPK in silicosis, we investigated the expression of AMPK and mTOR. Our study showed that the p-AMPK expression was decreased, and then the autophagy regulatory protein p-mTOR was activated in silica group (Figure 4A, Figure 8A). Up to now, the role of AMPK in silicosis has not been reported. However, in IPF, within the regions of active fibrosis, a significant decrease in AMPK activity was observed together with reduced activation of the Thr172 (Rangarajan et al., 2018). It is noted that AMPK is a positive regulator of autophagy while in our study silica exposure caused an augment of the autophagy activity. The observed autophagy activity induced by silica may be attributed to several factors including engulfment of pre-autophagosomal structure, impaired autophagic degradation by silica. In addition, pro-fibrogenic factors BCL-binding component 3 (BBC3) and monocyte chemoattractant protein-1-induced protein 1 (MCP1P1) during fibrotic process may also contribute to the silica-induced autophagy (Liu et al., 2016; Liu et al., 2017). These factors may override the regulatory effects of AMPK and promote autophagy activity by silica. However, this speculation need to be confirmed by more research work.

Drug design and development are extremely expensive and time consuming. Recent studies showed that a drug may have effects on different diseases. Therefore, identification of new use or repurposing of an approved drug is becoming more attractive approach in disease treatment (Pushpakom et al., 2019). Derived from galegine, a natural product from the plant Galega officinalis, metformin is a commonly prescribed drug to treat type 2 diabetes globally. Interestingly, recent studies have found that metformin can effectively reverse bleomycin-induced pulmonary fibrosis (Gamad et al., 2018). Although the causes of silicosis and idiopathic pulmonary fibrosis are different, they share similarities in the pathological changes of lung tissue and common mechanism of pulmonary fibrosis. We speculated that metformin may have beneficial effects on silicosis. Therefore, in this study we investigated the effects of metformin on silicosis using *in vivo* and *in vitro* models.

As a unique anti-diabetic drug, metformin usually does not cause hypoglycemia (Nasri and Rafieian-Kopaei, 2014). Cassano et al. (Cassano et al., 2020) demonstrated that compared with the control group receiving a normal diet of rats, there was no statistical difference in the effect of metformin on blood glucose levels. And Luo et al. (2021) reported that after a daily intragastric administration of 500 mg/kg metformin for 35 days, there was no changes in blood glucose in rats, compared with the control group. We treated the rats with metformin at 100, 200, and 400 mg/kg/day for another 28 days. The dosage selection of metformin was based on reported toxicity of metformin in rats (Quaile et al., 2010) and a previous study (Gamad et al., 2018). The dosage range equals to human effective dose of 16, 32, and 64 mg/kg/day, respectively, according to the published conversion method (Nair and Jacob, 2016). We then examined the various parameters associated with inflammatory responses and fibrosis and compared them with those among the rats with different treatment. After treatment with different doses of metformin, the weight of the rats markedly increased, and the lung coefficient decreased to varying degrees. Metformin treatment only did not show any effects on body weight and lung coefficient. These results suggested that metformin recovered the general adverse health effects induced by silica particles. After metformin treatment, inflammatory cells and nodules and collagen fibers in the lung tissues were significantly reduced (Figures 2A,B). Quantitative analysis indicated metformin induced a 20–25% reduction of inflammation infiltration and fibrosis and the effects showed a dose-response manner among the rats received metformin at different concentration. The result suggested that metformin may have a positive therapeutic effect on silicosis (Figures 2C,D) through alleviating the inflammation and fibrosis, the hallmark processes leading to silicosis. We then examined the pathways involved in these processed to explore the mechanisms underlying the effects of metformin.

Macrophages ingestion of silica and release inflammatory cytokines, such as TNF-α, IL-1, and TGF-β. These in turn provoke recruitment of inflammatory cells into the alveolar wall and alveolar epithelial surface, initiating alveolitis and inducing epithelial to mesenchymal transition (Mossman and Churg, 1998; Robledo and Mossman, 1999). Studies found that metformin attenuates PM2.5-induced inflammation (Gao et al., 2020) and inhibits TGF-β1-induced EMT (Yoshida et al., 2020). Our experimental results showed that after metformin treatment, the expression of pro-inflammatory factors TGF-β1, TNF-α, and IL-1β were significantly decreased (Figures 3A–C, Figure 6). This suggests that metformin alleviates the inflammatory response of silicosis through its anti-inflammatory effects. However, after metformin treatment, the protein expression of Vimentin and α-SMA was significantly down-regulated, while the E-Cad expression was up-regulated (Figures 3D,E, Figure 7). These results suggest that metformin may inhibit silica-mediated pulmonary fibrosis by inhibiting cellular pathways leading to EMT. In addition to fibrotic process, EMT has been implicated in cancer progression and metastasis. It is known that invasive properties and metastasis are controlled by EMT (Pearson, 2019). Cell invasion and metastasis are hallmarks of cancer development (Jiang et al., 2015). The inhibitive effect of metformin on EMT observed in this study suggest that metformin may have beneficial effect on cancer treatment. Indeed, metformin has been found to be able to inhibit the invasion and migration of various cancer cells (Chen X. et al., 2015; Chen et al., 2020).

Some evidence demonstrated that enhancing autophagy reduces silica-induced pulmonary fibrosis. MiR-326 inhibits inflammation and promotes autophagy activity to alleviate silica-induced pulmonary fibrosis (Xu T. et al., 2019). A study suggested that dioscin reduced silica-induced apoptosis and cytokine production by promoting autophagy, thereby exerted anti-fibrosis effects in silica-induced pulmonary fibrosis (Du et al., 2019). Rapamycin protects alveolar epithelial cells from apoptosis and attenuates silica-induced pulmonary fibrosis through the enhancement of autophagy in the mouse model (Zhao et al., 2019). Emerging evidence indicated that autophagy plays an import role in the beneficial effects of metformin (Bharath et al., 2020; Ren et al., 2020).Metformin attenuates lipopolysaccharide-induced epithelial cell senescence by activating autophagy (Wang et al., 2021). Bharath et al. (2020) showed that metformin enhances autophagy and normalizes mitochondrial function to alleviate aging-associated inflammation. Metformin alleviates oxidative stress and enhances autophagy in diabetic kidney disease (Ren et al., 2020). Metformin enhanced the autophagy as indicated by the up-regulated Beclin1 and LC3 and down-regulated the expression of p62 (Figure 4A). Immunohistochemistry results confirmed the increased expression of LC3 after treated with metformin (Figure 4B). The results from *in vitro* experiments in co-culture system also confirmed that the expression levels of LC3 and Beclin1 increased, and the expression of p62 decreased in HBECs (Figure 8A). In addition, we used GFP-LC3 cells to detect the accumulation of mature LC3 by which the GFP-LC3 signal becomes punctate (Bravo-San Pedro et al., 2017). The results from Ad-GFP-LC3 transfected cells demonstrated that metformin increased the number of GFP-LC3-foci (Figure 8B). Thus, the *in vivo* and *in vitro* studies confirmed the promoted autophagy by metformin.

Metformin was reported to activate AMPK. AMPK is a heterotrimeric complex consisting of an α catalytic subunit, scaffold protein *β* subunit, and regulatory *γ* non-catalytic subunit (Hardie et al., 2012). Metformin activates AMPK by increasing the phosphorylation of the catalytic a subunit at T172 (Shaw et al., 2005). Our results showed that after treatment with metformin, p-AMPK expression was significantly increased and p-mTOR expression decreased in silica stimulated rat lung tissues (Figure 4A). *In vitro* results showed that after 72 h of co-cultivation with metformin, it was also showed that the increased expression of p-AMPK and decreased p-mTOR compared with silica group. On the contrary, after the intervention of CC, the expression of p-AMPK protein was significantly down-regulated and the expression of p-mTOR was up-regulated in HBEC cells (Figure 8A). To confirm the involvement of AMPK/mTOR pathway, we treated cells with CC, an AMPK inhibitor, together with silica particles or silica plus metformin. So far, CC remains the only small molecule that has been widely used to study AMPK signaling and various aspects of cell physiology, including cell proliferation, survival, and migration (Liu et al., 2020). Yan et al. (Yan et al., 2010) found that inhibition of AMPKα activity either by CC or by RNA interference markedly reduced the accumulation of LC3-II. Chiou et al. (2020) indicated that compound C treatments reduced AMPKα1 mRNA levels, which resulted in suppressed AMPKα protein expression and AMPKα phosphorylation in CC-treated cells. It should be noted that although CC has been used as AMPK inhibitor for over a decade, its inhibitive effect of CC is not specific to AMPK (Liu et al., 2014) which means others kinase may be affected. The non-specific effects generally occur at relatively high concentration (over 5 µM) (Dasgupta and Seibel, 2018) and in this study we used 1 µM to minimize the non-specific effects. Treatment of CC led to 50% decrease of p-AMPK and about 10% increase of p-mTOR. As expected, CC caused a depression of autophagy by up regulating p62 and down regulating Beclin1 and LC3B. Meanwhile immunofluorescent staining showed that CC decreased the number of GFP-LC3-containing puncta and the protein LC3B levels in HBEC cells (Figure 8B:e). However, GFP-LC3 foci regained after application of metformin (Figure 8B:e). Thus, all *in vivo* and *in vitro* data indicated that metformin exert its anti-silicotic effects through AMPK/mTOR medicated autophagy.

Drug repurposing has gained more and more attractions in identifying new therapeutic way. Metformin as a threptic drug for type 2 diabetics has been used for 60 years. Recent studies found that metformin have benefits for other diseases including liver, heart and renal diseases as well as cancer, obesity and even aging (Lv and Guo, 2020). The findings in this study will undoubtedly has clinical utility as an additional therapeutic option for patients with silicosis especially for those co-existing with type 2 diabetes or diseases mentioned above.

## Conclusion

**FIGURE 9:**
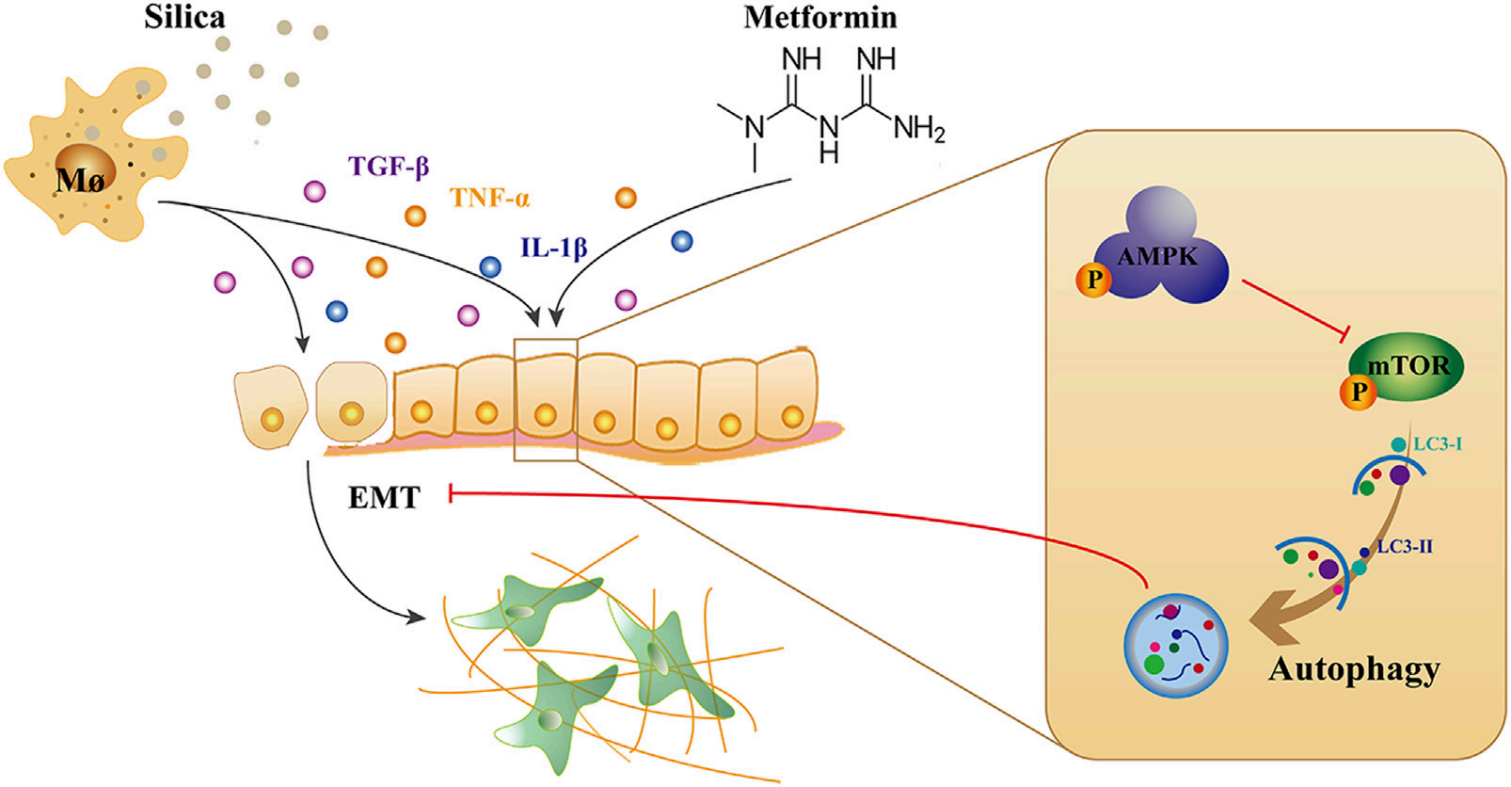
The mechanism of action of metformin against silica-induced pulmonary fibrosis. Uptake of silica by alveolar macrophages release high level of inflammatory cytokines, and induced EMT which epithelial cells gradually lose their epithelial characteristics and acquire the mesenchymal phenotype. Metformin can activate AMPK and inhibit mTOR, leading to autophagy induction, thereby reducing the EMT process in silicosis.

This study demonstrated that metformin has anti-silicotic potency in rats and *in vitro* cultured human cells. The effects of metformin may be due to its ability to alleviate the degree of alveolitis and pulmonary fibrosis, inhibit epithelial-mesenchymal transformation and alleviate the process of silica-induced pulmonary fibrosis. In addition, we showed that metformin can regulating autophagy through activating AMPK and inhibiting mTOR. Based on the results of our and others, we proposed the mechanism of action of metformin against silica particle-induced fibrosis as shown in Figure 9. The results from this study provide evidence that metformin may be potential therapeutic drug for effective treatment of silicosis.

## Data Availability

The original contributions presented in the study are included in the article/Supplementary Material, further inquiries can be directed to the corresponding authors.

## Supplementary Material

SUPPLEMENTARY FIGURE 1 The schematic diagram of the procedure of animal experiments and cells co-culture. **(A)** In vivo animal experiments, all rats were divided into six groups, the silica model group and three metformin treatment groups were injected with silica suspension into the lung once using a non-exposed tracheal intubation, and the rats in the negative control group and metformin control group were both injected with normal saline solution. After exposed to silica for 28 days, the rats were given a daily intragastric administration for 28 days, respectively. **(B)** In vitro cell experiment, THP-1 cells were placed into the insert, where they were treated by PMA. HBEC cells were cultured alone at the bottom. Then, the cell culture inserts containing THP-1 macrophages were transferred to the plates containing HBECs and received the indicated interventions for 72 h.
