# Supplementary figures and images for: Metformin Attenuates Silica-Induced Pulmonary Fibrosis by Activating Autophagy via the AMPK-mTOR Signaling Pathway
Source: Front Pharmacol. 2021 Aug 9;12:719589. doi: 10.3389/fphar.2021.719589 (PMC8381252; doi:10.3389/fphar.2021.719589)

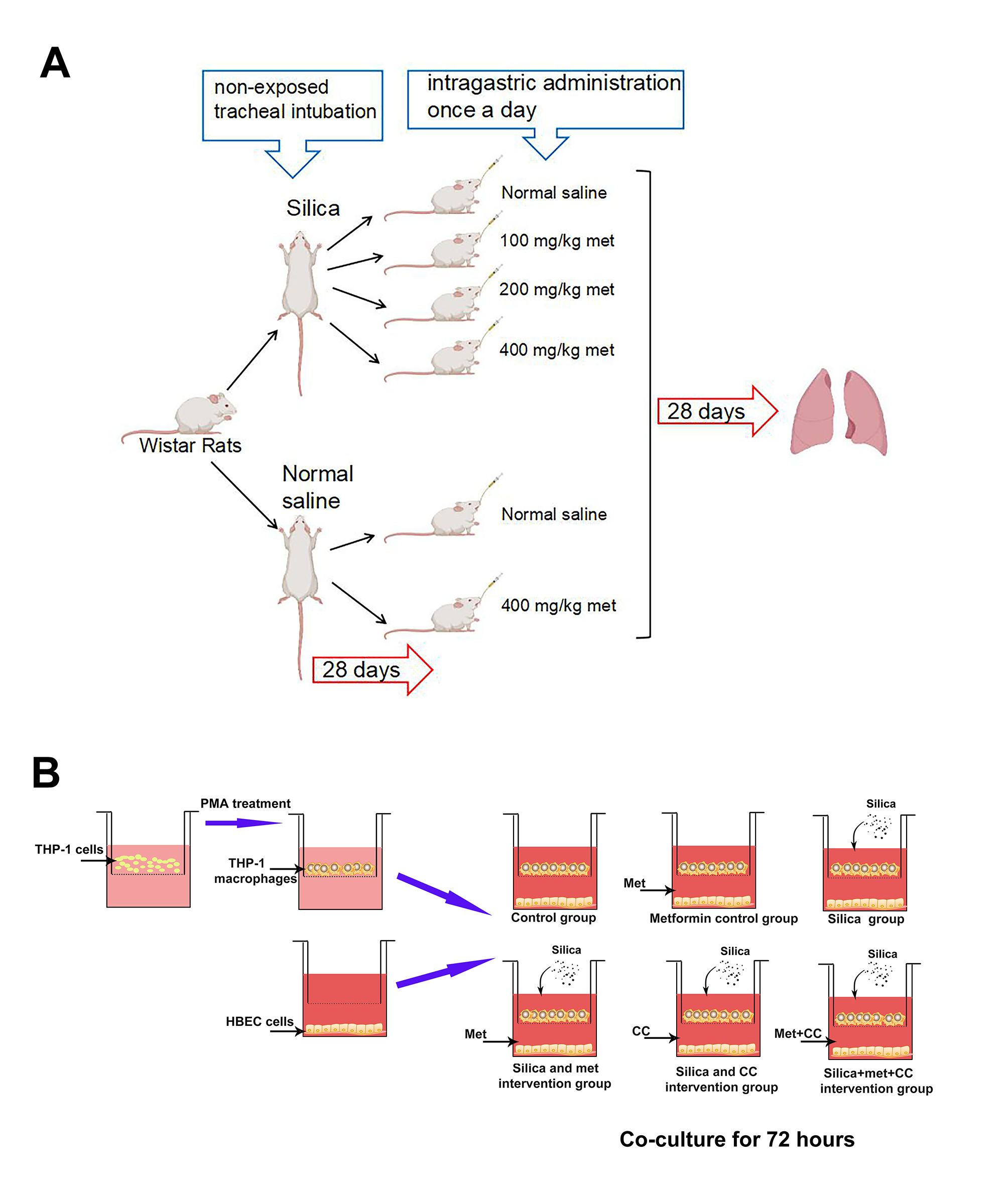

Supplement: Supplementary file 1 [file Image1.TIF]
